# Differential effects of a single dose of Lisdexamfetamine and Guanfacine on cognitive function in children with ADHD

**DOI:** 10.3389/fpsyt.2025.1676472

**Published:** 2025-11-03

**Authors:** Sahid El Masri, Olivia S. Kowalczyk, Tsz Hei Chiu, Marion Criaud, Steve Lukito, Ndabezinhle Mazibuko, Gonzalo Salazar de Pablo, Violeta Perez Rodriguez, Orsolya Makos, Sheut-Ling Lam, Samuel Westwood, Alexander Eaton-Turner, Natali Bozhilova, Aldo Conti, Paramala Santosh, Veit Roessner, Gregor Kohls, Mitul A. Mehta, Katya Rubia

**Affiliations:** 1 Department of Child & Adolescent Psychiatry, Institute of Psychiatry, Psychology & Neuroscience, King’s College London, London, United Kingdom; 2 Department of Child & Adolescent Psychiatry, Medical Faculty, Dresden University of Technology, Dresden, Germany; 3 Department of Neuroimaging, Institute of Psychiatry, Psychology & Neuroscience, King’s College London, London, United Kingdom; 4 Department of Child and Adolescent Psychiatry, Institute of Psychiatry and Mental Health, Hospital General Universitario Gregorio Marañón, Instituto de Investigación Sanitaria Gregorio Marañón (IiSGM), Centro de Investigación Biomédica en Red de Salud Mental (CIBERSAM), Instituto de Salud Carlos III (ISCIII), School of Medicine, Universidad Complutense, Madrid, Spain; 5 Department of Psychosis Studies, Institute of Psychiatry, Psychology & Neuroscience, King’s College London, London, United Kingdom; 6 Department of Child & Adolescent Psychiatry, Medical Faculty, Dresden University of Technology, German Center for Child and Adolescent Health (DZKJ), partner site Leipzig/Dresden, Dresden, Germany

**Keywords:** ADHD (Attention-Deficit/Hyperactivity Disorder), lisdexamfetamine, guanfacine, mean reaction time (MRT), reaction time variability (RTV), sustained attention, continuous performance task, time estimation

## Abstract

**Background:**

Attention-Deficit/Hyperactivity Disorder (ADHD) is associated with cognitive difficulties which improve with traditional stimulant and non-stimulant medications. However, there is limited evidence on the cognitive effects of the newer licensed stimulant lisdexamfetamine and non-stimulant guanfacine in children with ADHD. Therefore, we compared differential single-dose effects of lisdexamfetamine and guanfacine on cognitive performance in youth with ADHD.

**Methods:**

In a randomized, placebo-controlled, double-blind, cross-over design, 22 children with ADHD were tested in tasks of sustained attention, vigilance, motor and interference inhibition, and time discrimination after single doses of guanfacine extended release, lisdexamfetamine, and placebo, with weekly washouts. Across tasks, composite measures of mean reaction time (MRT), intra-subject reaction time variability (coefficient of variation; CV), thought to reflect inattention, and premature responses were analyzed. Age-, IQ-, and sex- matched typically developing youth were assessed once without medication to test for potential drug normalization effects on performance differences compared to participants with ADHD on placebo.

**Results:**

Lisdexamfetamine significantly improved MRT and CV, while guanfacine worsened CV, compared with placebo and the other drug, with large effects. Although not reaching significance, there were moderate to large effects for lisdexamfetamine improving time discrimination and omission errors and for guanfacine to worsen omission errors in a sustained attention task relative to placebo and the other drug.

**Conclusion:**

These differential effects of lisdexamfetamine improving MRT and CV, while guanfacine worsening CV are clinically relevant, because they are the most replicated cognitive impairments in youth with ADHD. Findings suggest that guanfacine, unlike lisdexamfetamine, may not improve attention in children and adolescents with ADHD.

**Clinical Trial Registration:**

https://clinicaltrials.gov/study/NCT03333668?term=NCT03333668&rank=1#study-plan, identifier NCT03333668.

## 1 Introduction

Attention-Deficit/Hyperactivity Disorder (ADHD) is the most common neurodevelopmental condition affecting up to eight percent of children worldwide (1). ADHD is defined by the Diagnostic and Statistical Manual of Mental Disorders (DSM)-5 as persistent, developmentally-inappropriate symptoms of inattention and/or hyperactivity/impulsiveness (2). ADHD is associated with comorbidities and poor social and academic outcomes (3). Meta-analyses and systematic reviews have furthermore shown difficulties in children and adults with ADHD relative to those without ADHD in cognitive task performance, most consistently in executive functions, specifically in motor/interference inhibition (4, 5), working memory (WM) (6, 7), sustained attention (8) and timing (9, 10), although there is heterogeneity, with some individuals not showing differences relative to typically developing (TD) controls (11). Furthermore, there is consistent evidence for slower processing speed and the most replicated cognitive difference is a more variable response style reflected in increased intra-subject reaction time variability (RTV) (12–17), which is associated with ADHD symptoms (18) and problems of attention, state regulation (i.e. arousal), mind-wandering and motor timing (13, 17, 19–21).

It is paramount to test the effects of ADHD medications on cognitive performance and not just diagnostic symptoms, given poor correlation between them (22) and the impact cognitive problems have on academic (23) and professional life (24).

First line treatment for severe ADHD is stimulant medication which increases dopamine and norepinephrine, most commonly methylphenidate, followed by (dex)amphetamine and lisdexamfetamine, showing large effect sizes of 0.78 for methylphenidate and 0.85 for amphetamines based on clinician ratings in reducing clinical symptoms in about 70% of children with ADHD (3, 25). Unlike traditional stimulants, the prodrug lisdexamfetamine releases active dextroamphetamine only after being metabolized in the body, making it one of the longest-acting stimulants for ADHD with effects lasting for 13 to 14 hours after ingestion (26).

Second-line treatment is with non-stimulant medications such as selective norepinephrine reuptake inhibitor atomoxetine which reduces clinical symptoms with medium effect sizes (0.45) in children with ADHD (25). The newer licensed non-stimulant guanfacine which is an α2-adrenergic receptor agonist reduces ADHD symptoms with medium effect-sizes (0.5) in children with ADHD (25). Guanfacine also has mild sedative properties (27), potentially underlying its positive impact on behavioral ADHD symptoms, especially hyperactivity (28). However, the tolerability of guanfacine in children with ADHD is poor. It has the most drop-outs due to side effects of all tested medications according to a network meta-analysis, closely followed by amphetamine-based medication such as lisdexamfetamine (25).

Four meta-analyses testing predominantly single dose effects of stimulants on cognitive performance in children and adults with ADHD, have most consistently found effects on mean reaction time (MRT), attention, inhibition and WM (17, 22, 29, 30). Our meta-analysis showed that longer-term administration of both methylphenidate and atomoxetine improved MRT, attention and inhibition with statistically comparable small to medium effect sizes (31).

Few studies, however, tested cognitive effects of the newer licensed ADHD drugs lisdexamfetamine or guanfacine. Meta-analyses have shown efficacy of lisdexamfetamine in improving ADHD symptoms with large effect sizes (32, 33). A meta-analysis examining efficacy and tolerability of several ADHD drugs on behavioral symptoms, furthermore, showed a higher effect size for lisdexamfetamine than for methylphenidate in improving symptoms in both children and adults with ADHD and recommended lisdexamfetamine as first line treatment next to methylphenidate in adults with ADHD, but not in youth because of higher dropouts due to side effects (25).

So far, only one open-label and four randomized placebo-controlled studies tested the effects of longer-term administration of lisdexamfetamine on cognition in ADHD including two functional magnetic resonance imaging (fMRI) studies. The open label study showed that two years of lisdexamfetamine compared to baseline improved attention, short-term visual memory, response inhibition and WM in 191 children with ADHD (34). In 17 adults with ADHD, seven days of lisdexamfetamine relative to placebo improved MRT and vigilance (35). Five weeks of lisdexamfetamine in 24 adults with ADHD improved memory, RTV and commission errors in the continuous performance task (CPT) relative to placebo, but did not improve MRT or omission errors relative to placebo (36). Two fMRI studies showed no performance improvements in decision making in 20 adults with ADHD after three to five weeks daily intake of lisdexamfetamine relative to placebo (37), nor after three to four weeks of lisdexamfetamine treatment in an emotional Go/No-Go task in 25 adults with ADHD (38).

Only six randomized placebo-controlled studies tested the effects of mostly longer-term use of guanfacine on cognitive performance in people with ADHD. These include four studies measuring brain mechanisms (fMRI, functional near-infrared spectroscopy [fNIRS] and electroencephalogram [EEG]) and two studies including a stimulant as comparison. The majority of studies found no effects of guanfacine on cognition; eight weeks of guanfacine compared to placebo did not improve WM performance, response inhibition, MRT, or RTV in 182 children with ADHD (39); likewise, 45 days of guanfacine relative to placebo had no effect on RT, sustained attention, spatial WM, and visual-motor processing in 178 children with ADHD (40). Two imaging studies found no effects of guanfacine on Go/No-Go task performance in 25 children with ADHD during fMRI (41), nor after a single dose of guanfacine relative to placebo in 12 children with ADHD during fNIRS (42). An EEG study found that, after eight weeks of guanfacine, spatial WM performance worsened in 172 children with ADHD compared to both placebo and d-methylphenidate (43), and only the combination of both drugs improved spatial WM. Only one small study of 17 adults with ADHD showed improvement in focused attention and interference inhibition in the Stroop task after two weeks of both guanfacine and dextroamphetamine relative to placebo, with guanfacine being superior to dextroamphetamine (44).

In summary, most studies tested cognitive effects of relatively longer-term administration of lisdexamfetamine and guanfacine against placebo, and only two studies compared guanfacine with a stimulant (methylphenidate and dextroamphetamine), but no study has directly compared the effects of lisdexamfetamine and guanfacine in individuals with ADHD. While testing longer-term effects on cognition is clinically relevant, testing single dose effects is also valuable because they could be less confounded by longer-term purely developmental effects, side effects and clinical improvements that could indirectly affect cognition (45).

The aim of this study was therefore to compare in children with ADHD in a randomized placebo-controlled, double-blind, crossover design the differential effects of single doses of guanfacine extended release (GXR) and lisdexamfetamine relative to placebo on performance on a range of ADHD-relevant cognitive functions such as sustained attention, vigilance, motor, interference inhibition and time discrimination as well as composite measures of MRT and RTV across tasks. Based on previous findings (34–40, 43, 44), we hypothesized that performance would be exclusively or more improved by lisdexamfetamine relative to placebo and guanfacine in all measures except for inhibition measures which would be equally improved under both drugs relative to placebo (44).

## 2 Methods

### 2.1 Study design

This study was preregistered on ClinicalTrials.gov (Identifier: NCT03333668) before data collection commenced. The full preregistration can be accessed at ClinicalTrials.gov: *https://clinicaltrials.gov/study/nct03333668*.

This study was an experimental pharmacological study testing the effects of a single clinical dose of either lisdexamfetamine, guanfacine and placebo on a range of cognitive functions and on brain activation in fMRI, in children and adolescents with ADHD, in a within-group, counterbalanced, 1-week washout, repeated measures design.

### 2.2 Participants

Twenty-two children and adolescents (female = 6) between 8 and 20 years of age (mean age = 13.5, standard deviation [SD] = 3.1) meeting diagnostic criteria for predominantly inattentive presentation, predominantly hyperactive-impulsive presentation or combined presentation of ADHD based on the DSM-5 were recruited from NHS Trusts, private clinics in (Greater) London, social media, ADHD and parent support groups and a participant data bank from another study. Participants were either medication-naïve or willing to stop taking stimulant medications for at least 48 hours prior to each of the three testing sessions or for at least two weeks for any other psychoactive medications. Participants first came for an in-person pre-assessment, including administration of an electrocardiogram (ECG), measurements of blood pressure and body weight, an intelligence test (IQ) using the WASI-II (46) and a familiarization with MRI scanner environment in a mock scanner since the main goal of the project was to examine the neural correlates of the drugs. Inclusion required a score above clinical cut-offs in the ADHD Rating Scale (ADHD-RS (47); (total score of 24/90^th^ percentile), Conners Parent Rating Scale (CPRS-R (48); (DSM-IV-TR criteria; >6 symptoms for either DSM-5 inattention or hyperactivity or both) and the Kiddie Schedule for Affective Disorders and Schizophrenia (K-SADS (49); (DSM-5 criteria; six or more symptoms for either DSM-5 inattention or hyperactivity or both). Participants were excluded if they had an IQ below 70, MRI contraindications (e.g. metallic implants, previous surgical procedures, a history of migraine, braces, claustrophobia), a history of substance abuse, neurological (e.g., brain tumor, epilepsy or a history of symptomatic seizures, polyneuropathy) or any other major clinical psychiatric disorder except for mild mood and depression symptoms, autism, phobias, anxiety, conduct disorder, oppositional defiant (ODD) or eating disorders. Also excluded were learning disability, reading, speech, or language disorder or other somatic illnesses (cardiovascular, renal, hepatic, metabolic) that would impact the data integrity or safety of the subject (i.e. contraindications to any of the treatments) as determined by the investigators. Participants also completed the Edinburgh Handedness Inventory (50). Moreover, 24 sex, IQ and age-matched TD control subjects (mean age = 14.3, SD = 3.1), were tested once without medication to examine potential normalization effects of the two drugs on cognitive differences between participants with ADHD under placebo and TD participants. Inclusion criteria for the TD participants were an IQ above 70, a score below the cut-off (i.e., 13) for autistic traits on the Social Communication Questionnaire (SCQ) (51) and for ADHD on the CPRS-R (6 symptoms for either DSM-5 inattention or hyperactivity or both), no history of substance abuse and no neurological deficits, learning disability, reading, speech, language disorder or other illness that would impact the data integrity or safety of the subject as determined by the investigators as well as no MRI contraindications. Compensation was £20 for the pre-assessment visit and £50 for each testing session. Parental/carer/child written informed consent/assent and approval from the London Bridge Research Ethics Committee (REC; 18/LO/0472) were obtained.

### 2.3 Testing procedure

Participants with ADHD attended three study visits, on which they received one single clinical dose of either guanfacine, lisdexamfetamine or placebo, in a counterbalanced way, with at least seven days between each visit to allow for medication washout. GXR has a half-life of approximately 16 hours (27) and lisdexamfetamine of approximately 9 hours. Calculating washout period as 6.5 times the half-life of each drug, seven days was chosen as both pragmatic and sufficient to avoid drug interactions. On each visit, participants with ADHD received either a placebo or a single, typical clinical oral doses of lisdexamfetamine or GXR respectively, all over-encapsulated in matching capsules by the Maudsley pharmacy. Two participants experienced transient mild adverse events after lisdexamfetamine. Thereafter, the original doses of lisdexamfetamine (30mg for participants under 30kg of body weight, 50mg for participants over 30kg of body weight) were adjusted: 8- and 9-year-old participants with ADHD received 20mg regardless of weight and for participants aged 10–20 years the dose was 0.5 mg/kg, rounded down to the nearest 30mg, 40mg, or 50mg, with intermediate doses rounded down to the closest 10mg (e.g., 35mg to 30mg). Guanfacine was given at 0.05 mg/kg and rounded down to the nearest 1 mg, 2 mg, or a maximum of 3 mg. Placebo contained 10 mg vitamin C.

A computer-generated randomization list was given to the Maudsley pharmacy for over-encapsulation, blinding and dispensing of the capsules. Neither the researcher nor the participants and parents/carers were aware of dosing information throughout data acquisition. Parents/carers were unblinded at the end of recruitment by the senior researcher. The recruiting researcher was unblinded after recruitment ended and the first blinded data analysis was completed.

There was a discrepancy between the time to peak drug concentration (t-max) of lisdexamfetamine (3.5 – 4.5h) and guanfacine (5h) and since the schedule was mainly tailored toward the fMRI (4.5h after medication administration), the neurocognitive testing was compromised to be at three hours after drug administration. After each of the three in-person visits, any adverse effects were recorded, using the adapted adverse effects questionnaire for parents (52).

The neurocognitive task battery included tasks of the Maudsley Attention and Response Suppression (MARS) task battery (53, 54) and an adaptation of the Mackworth Clock Vigilance task (MCT) (55). The MARS task battery included the Go/No-Go task, the Simon interference inhibition task (Simon task), the CPT and a time discrimination task. The Go/No-Go task is a selective motor response inhibition task. Participants are instructed to give motor responses as fast as possible to high frequent Go signals (300ms duration; 70%) and inhibit motor responses to infrequent No-Go signals (300ms duration; 30%). The dependent variable was the probability (%) of inhibition to the No-Go stimuli. The Simon task measures interference inhibition. Participants have to give corresponding (left/right) motor responses to left/right arrows presented on the screen on the left or right side (72.7% congruent trials; 300ms duration), but overwrite prepotent response tendencies to incorrectly respond to spatially incompatible cues (left/right pointing arrows appearing on right/left side of screen; 27.3% incongruent trials). The dependent variable is the Simon RT interference effect (MRT to congruent trials subtracted from MRT to incongruent trials). The CPT measures sustained and selective attention. Participants are presented (300ms duration) with letters (A to L), which they have to ignore, except the specific target letters (“A” followed by “X” or “A” followed by “O”), to which they have to respond with left and right motor responses on a keyboard, respectively. Dependent variables are the percentage of omission errors (i.e., missed detection to target trials, reflecting inattention) and commission errors (incorrect response to nontarget items, i.e., false hits, reflecting impulsiveness (56)). The Time Discrimination task measures the ability to discriminate between time intervals that differ by several hundreds of milliseconds. One green or red circle (e.g. red) is randomly presented for a standard duration of 1000ms and a second circle (e.g. green) for either 1300, 1400, or 1500ms. After presentation of both circles, participants need to indicate which circle presented longer on the screen by giving a left/right motor response. To ensure a consistent strategy, participants were asked to discriminate the time intervals by counting silently. The dependent variable is the percentage of time discrimination errors. The MCT measures sustained attention and vigilance on the detection of signals that are difficult to identify. This task presents a clock-hand in the middle of the screen, rotating clockwise every second like a real clock. In 10% of 400 trials the clock-hand double jumps (i.e. skipping a second). Participants need to give motor responses within 1s, if they thought they saw the double jump. Participants receive an error signal (pivot turning red) when a response was made in the absence of double jumps or when they fail to detect them and a green light when the response is correct (i.e., they pressed to a double-jump). Dependent variables are the percentage of omission and commission errors. For more detailed information and schematic displays of the tasks, see supplemental data (A).

Across the Go/No-Go, Simon and CPT tasks composite scores for MRT, CV and premature responses (PREM) were calculated, based on our previous publication (57). The CV is a measure of intraindividual variability of RT that controls for differences in RT (58). Premature responses reflect behavioral impulsiveness in ADHD and are defined as responses made 200ms before stimulus and 100ms after the stimulus (as this is too short to be a RT) (54). For the calculation formulas of the composite scores see (57) supplemental data (B). Overall, we tested 10 cognitive variables: Go/No-Go probability of inhibition (Go/No-Go PI) (%), Simon RT effect (ms), CPT omission and commission errors (%), MCT omission and commission errors (%), Time Discrimination errors (%), and the composite measures of MRT (ms), CV and (PREM) (%) across the Go/No-Go, CPT and Simon tasks.

### 2.4 Statistical analysis

Repeated-measures analysis of variance (ANOVA) was conducted to examine the effects of the three drug conditions (lisdexamfetamine, guanfacine and placebo) on the 10 cognitive variables. We also tested for differences of the continuous score of total amounts of reported adverse effects. Because guanfacine can cause drowsiness we also specifically tested the ordinal scale adverse effect items drowsiness and fatigue given known effects of guanfacine on these measures (27), using Friedman tests. *Post-hoc* t-test group comparisons of means were calculated to assess direction of significant effects, and false discovery rate (FDR) was used for correcting multiple testing.

To explore potential drug normalization effects, we conducted a series of t-tests comparing the ADHD group on placebo and on each drug condition with the TD group. These analyses were limited to cognitive variables that showed significant within-group effects of drug condition in the ADHD group.

For the TD group, to identify statistical outliers, interquartile ranges (IQR) were calculated for all cognitive variables; two participants were excluded from analysis because they were outliers (minimum 1.5 IQR) in their performance in a minimum of three measures, leaving the TD group with 22 subjects (mean age = 14.3, SD = 3.1). Because 50% of participants with ADHD had a diagnosis of ASD and scored high on the SCQ (>12), we included regression analyses to examine the potential impact of the ASD diagnosis and high ASD traits in the SCQ.

To also indirectly test order effects for the composite measures, we used Go/No-Go RT to Go signals, Simon RT to congruent signals, and CPT MRT to targets instead of the composite MRT across tasks, Go/No-Go SD of RT to Go signals, Simon SD of RTs to congruent signals, and CPT intrasubject SD of MRT for all targets instead of CV across tasks, and premature responses to all Go/No-Go, Simon, and CPT signals (%) instead of premature responses across tasks.

Two participants with ADHD chewed the capsules containing lisdexamfetamine, guanfacine and placebo, instead of swallowing them. GXR should not be crushed or chewed as this increases the rate of guanfacine release (27). However, IQR of performance showed that only one of the two participants who chewed guanfacine was an outlier in just one of the 10 measures (MCT COM). Nonetheless, all analyses were repeated without the two participants to ensure validity of the results.

To assess normal distribution of the 10 cognitive variables, the variable for the total amount of reported adverse effects and the adverse effects questionnaire items drowsiness and fatigue, Shapiro-Wilk tests were conducted for the ADHD group and they showed non-normality on at least one out of three medication conditions in each of the 10 cognitive variables and in the variables for total adverse effects (p<0.05). ANOVA is robust against violations of the normality assumption (59); however to ensure validity of the results, we also conducted nonparametric tests. Statistical analyses were performed using IBM SPSS Statistics (Version 28.0.1.1).

## 3 Results

### 3.1 Demographic data

For baseline characteristics, as expected, t-tests showed significant differences in the scores for CPRS and SCQ between the ADHD and TD groups (p<0.001). No other differences were observed in any other measures (see **Table 1**).

**Table 1:** Demographic and clinical data in participants with ADHD and TD.

	ADHD (N = 22) mean (SD)	TD (N = 22) mean (SD)	T-value / Pearson-χ²	df	P value
**Sex (%)**			χ²= 0.00*^2^	1*^2^	1.0*^2^
Male	16 (72.7)	17 (77.3)			
Female	6 (27.3)	5 (22.7)			
**Age in years**	14.0 (3.0)	15.1 (3.4)	t= 1.13	42	0.265
**Handedness (%)**			χ²= 2.04	2	0.360
Left	4 (18.2)	1 (4.5)			
Right	17 (77.3)	20 (91)			
No preference	1 (4.5)	1 (4.5)			
**Full scale IQ**	107.1 (15.4)	110.3 (12.5)	t= 0.76	42	0.450
CPRS-R					
DSM-IV Inattention t	83.5 (8.6)	46.3 (5.7)	t= -16.91	42	<0.001
DSM-IV Hyperactivity t	82 (10.4)	48.8 (6.9)	t= -12.48*^1^	36.3*^1^	<0.001
ADHD-RS					
Total	39.7 (8.9)				
Inattention	22.7 (3.6)				
Hyperactivity	17.1 (5.8)				
**ADHD clinical diagnosis (pre-existing diagnosis through CAMHS) (%)**	17 (77.3)				
**ODD clinical diagnosis (pre-existing diagnosis through CAMHS) (%)**	1 (4.5)				
K-SADS-based research diagnosis (%)					
Inattentive type	5 (22.7)				
Hyperactive type	0 (0)				
Combined type	17 (77.3)				
ODD	8 (36.4)				
**ASD clinical diagnosis (pre-existing diagnosis through CAMHS) (%)**	10 (45.5)				
**Medication naive (%)**	11 (50)				

Significant at p = 0.05; ADHD, Attention-Deficit/Hyperactivity Disorder; TD, typically developing controls; SD, standard deviation; df, degrees of freedom;*1, Equal variances not assumed (welch-corrected); *2, yates-corrected (continuity correction; 2×2 tables only); IQ, intelligence quotient; CPRS-R, Conners’ Parent Rating Scale–Revised; DSM, Diagnostic and Statistical Manual of Mental Disorders; ADHD-R, Attention-Deficit/Hyperactivity Disorder Rating Scale; ODD, oppositional defiant disorder; K-SADS, Kiddie Schedule for Affective Disorders and Schizophrenia; ASD, autism spectrum disorder; CAMHS, Child and Adolescent Mental Health Services.

### 3.2 Test of within-subject medication effects on cognitive performance in the ADHD group

**Figure 1:**
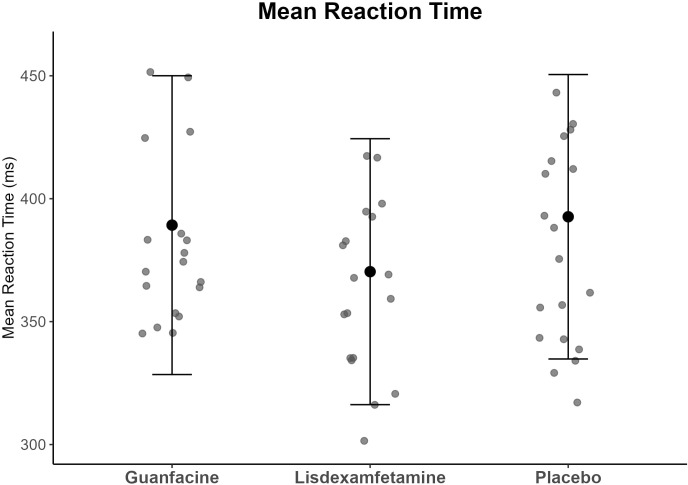
Means and standard deviation of the MRT composite scores for each drug condition. Mean (point) ± 1 SD (error bars); ms= milliseconds;.

**Figure 2:**
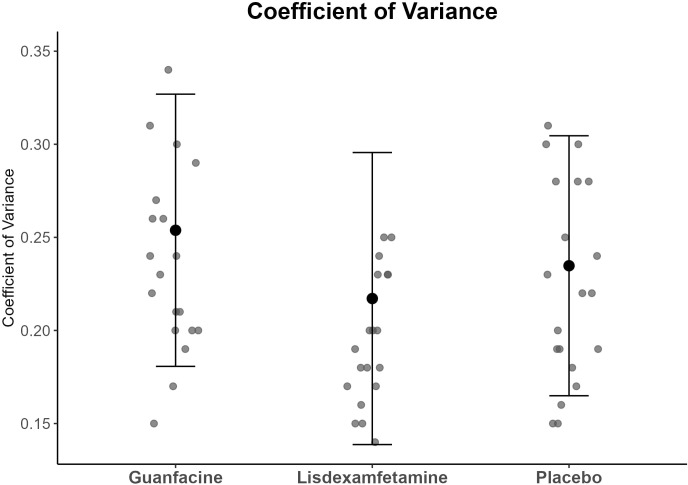
Means and standard deviation of the CV composite scores for each drug condition. Mean (point) ± 1 SD (error bars).

ANOVAs showed significant main effects of drug for MRT (p <0.001, ES = 1.31) (see **Figure 1**) and CV (p <0.001, ES = 1.34) with large effect sizes (see **Figure 2**) (for means and SDs see **Table 2**). *Post-hoc* t-tests (df = 20) showed that youth with ADHD under lisdexamfetamine had faster MRT compared to placebo (t= -3.69, p= 0.003) and guanfacine (t= -3.31, p= 0.003) and lower CV compared to placebo (t= -1.78, p= 0.045) and guanfacine (t= -5.31, p= 0.031), while guanfacine worsened CV relative to placebo (t= 2.17, p= 0.031); (all p-values were FDR-corrected).

**Table 2:** Performance data and within group (ADHD) drug differences.

Cognitive variables	N	TD control subjects mean (SD)	N	ADHD placebo mean (SD)	ADHD lisdexamfetamine mean (SD)	ADHD guanfacine mean (SD)	F	df	p value	Cohen’s d
Go/no-go PI (%)	22	61.08 (14.80)	22	66.07 (19.01)	65.05 (22.02)	60.11 (22.63)	2.22	2	0.121	0.65
Simon RT interference effect (ms)	22	71.11 (51.38)	22	52.94 (33.79)	56.83 (29.73)	53.38 (26.16)	0.15	2	0.860	0.17
CPT omission errors (%)	22	6.82 (6.15)	21	8.70 (8.70)	6.82 (7.83)	11.98 (10.80)	4.30	1.64*^1^	0.054*^1^	0.82
CPT commission errors (%)	22	0.61 (0.67)	21	0.87 (0.96)	0.96 (1.11)	1.53 (2.69)	1.38	1.27*^2^	0.259*^2^	0.53
MCT omission errors (%)	20	20.88 (13.16)	20	27.25 (20.55)	22.12 (18.03)	28.00 (20.30)	1.20	2	0.311	0.50
MCT commission errors	20	1.60 (1.21)	20	1.98 (1.88)	1.86 (1.74)	2.48 (2.12)	1.19	2	0.313	0.50
Time discrimination errors (%)	21	17.94 (15.16)	22	28,11 (23.63)	20.98 (17.23)	26.81 (19.53)	2.68	2	0.080	0.72
MRT composite score (ms)	22	364.04 (38.00)	21	392.65 (57.84)	370.31 (54.08)	389.24 (60.77)	8.61	2	<0.001	1.31
CV composite score	22	0.23 (0.44)	21	0.23 (0.69)	0.21 (0.78)	0.25 (0.72)	9.00	2	<0.001	1.34
PREM composite score	22	0.43 (0.44)	21	0.53 (1.07)	0.59 (1.50)	0.76 (1.42)	0.91	1.19*^2^	0.367*^2^	0.42

Significant at p = 0.05; PI, Probability of inhibition; RT, Reaction time; CPT, Continouts Performance Task; MCT, Mackworth Clock Task; MRT, Mean reaction time; CV, Coefficient of variation; PREM, Premature responses; *^1^Huynh-Feldt-Correction; *^2^Greenhouse-Geisser correction.

The ANOVA also showed two medium to large sized effects that did not reach the threshold of significance in time discrimination errors (p= 0.080, ES = 0.72) and CPT omission errors (p= 0.054, ES = 0.82). Descriptively mean differences suggested improved time discrimination and CPT omission errors under lisdexamfetamine and worsening of CPT omission errors under guanfacine. Exploratory FDR-corrected *post-hoc* t-tests showed the same tendency, with lisdexamfetamine improving time discrimination compared to both placebo (t = –1.869, p = .078) and guanfacine (t = –1.695, p = .078), and reducing CPT omission errors compared to placebo (t = –1.308, p = .103) and guanfacine (t = –2.122, p = .069), while guanfacine tended to increase CPT omission errors relative to placebo (t = 1.632, p = .089).

### 3.3 Test for normalization effects of cognitive performance relative to TD in those variables that showed a drug effect in the ADHD group

FDR corrected t-tests (df= 41) between the ADHD group on placebo and the TD in the cognitive variables that were significantly improved/worsened in the repeated-measures ANOVA (MRT, CV) showed no significant differences neither in MRT (t= 1.93, p= 0.093) nor in CV (t= 0.173, p= 0.432). Therefore, no further testing of potential normalization was conducted.

### 3.4 Adverse effects questionnaire

Frequencies of adverse effects reported by parents/carers of the participants with ADHD are shown in **Table 3**. ANOVA showed significant effects for total adverse effects (F = 14.86 df= 1.29, p<0.001), but Friedman tests for the single ordinal-scale items drowsiness (χ²= 2.51, df= 2, p= 0.285) or fatigue (χ²= 2.68, df= 2, p= 0.262) were not significant. *Post-hoc* t-tests showed that parents/carers of youth with ADHD (df=21) reported more total adverse effects in their children following lisdexamfetamine relative to placebo (t= 4.66, p= 0.002) and relative to guanfacine (t= 3.48, p= 0.002), while guanfacine and placebo did not differ (t= 1.31, p= 0.102) (all p-values were FDR-corrected).

**Table 3:** Adverse effects reported by carers of participants with ADHD (N = 21).

Reported adverse effect (%)	Lisdexamfetamine (N = 21)	Guanfacine (N = 21)	Placebo (N = 21)
Fatigued	7 (35.0)	8 (40.0)	3 (15.0)
Poor appetite	13 (65.0)	3 (15.0)	2 (10.0)
Drowsy	7 (35.0)	5 (25.0)	3 (15.0)
Has trouble getting off to sleep	10 (50.0)	1 (5.0)	2 (10.0)
Irritable mood	7 (35.0)	1 (5.0)	2 (10.0)
Complains of headache	6 (30.0)	2 (10.0)	2 (10.0)
Complains of stomachache	5 (25.0)	4 (20.0)	1 (5.0)
Excited	4 (20.0)	1 (5.0)	5 (25.0)
Stares a lot or daydreams	5 (25.0)	3 (15.0)	1 (5.0)
Easily distracted	2 (10.0)	2 (10.0)	4 (20.0)
Talks less than usual with other children/peers	4 (20.0)	3 (15.0)	0 (0.0)
Seems unsteady	3 (15.0)	2 (10.0)	0 (0.0)
Dizziness	3 (15.0)	1 (5.0)	0 (0.0)
Looks sad, miserable	3 (15.0)	1 (5.0)	0 (0.0)
Angry	3 (15.0)	1 (5.0)	0 (0.0)
Flushing	2 (10.0)	0 (0.0)	1 (5.0)
Not interested in other children/peers	1 (5.0)	1 (5.0)	1 (5.0)
Complains about light headedness and muscular weakness	3 (15.0)	0 (0.0)	0 (0.0)
Complains about dry mouth	1 (5.0)	1 (5.0)	1 (5.0)
Complains about palpitation	1 (5.0)	1 (5.0)	0 (0.0)
Looks anxious	2 (10.0)	0 (0.0)	0 (0.0)
Low confidence	1 (5.0)	1 (5.0)	0 (0.0)
Displays twitches (tics)	1 (5.0)	0 (0.0)	0 (0.0)
Complains about constipation	1 (5.0)	0 (0.0)	0 (0.0)
Crying spells	1 (5.0)	0 (0.0)	0 (0.0)
Has nightmares	0 (0.0)	0 (0.0)	0 (0.0)
Scratches himself /herself more, bites his/her nails or his/her lips more	0 (0.0)	0 (0.0)	0 (0.0)

### 3.5 Controlling for ASD diagnosis and high ASD traits

For MRT and CV, a regression model with separate predictors using either ASD diagnosis, or the continuous score of the SCQ did not significantly improve the explained variance in MRT (p> 0.05) or CV (p> 0.05) across any drug condition and none of the unstandardized coefficients for ASD diagnosis were significant (p> 0.05).

The increase in R², when the two different predictors for ASD were tested separately, was small across all drug conditions in both MRT and CV (max R² = 11.5%), i.e. ASD diagnosis or ASD traits were accounting for only a small proportion of the variance in the whole model of drug effects on MRT or CV.

### 3.6 Controlling for order effects

The within-subject ANOVA including order as between-subject-factor showed no significant main effects for order in RT or intrasubject SDRT in any of the tasks tested. Significant main effects for order were only found in CPT commission errors (F (5, 15) = 2.57, p = 0.030, partial η² = 0.531) and MCT omission errors (F (5, 15) = 3.39, p = 0.030, partial η² = 0.487), which were not affected by either drug in the within-subject ANOVA.

### 3.7 Controlling for non-normal distributed data and two children chewing guanfacine capsules

Findings did not change substantially when non-normal distributed data was analyzed with non-parametric tests (see supplemental data C) or when the two participants who chewed capsules of guanfacine were excluded from all analyses (see supplemental data D-E).

## 4 Discussion

This is the first study directly comparing the differential effects of single doses of lisdexamfetamine and guanfacine relative to placebo on cognitive performance in children with ADHD. Lisdexamfetamine significantly improved MRT and CV compared to guanfacine and placebo, while guanfacine worsened CV relative to placebo and lisdexamfetamine, with large effect sizes. We also showed medication effects that were slightly below the threshold of significance, but with medium to large effect sizes of lisdexamfetamine reducing time discrimination and CPT omission errors compared to placebo and guanfacine, and guanfacine worsening CPT omission errors relative to placebo (and lisdexamfetamine). Because the negative effects of guanfacine on cognition were opposite to our initial hypothesis, we repeated the analyses using two-tailed tests showing that non-parametric analyses confirmed the same pattern of effects.

The findings of improved RT and CV under lisdexamfetamine relative to guanfacine and placebo and in particular the deterioration of CV under guanfacine relative to placebo are highly relevant because slower and in particular more variable processing speed are among the most consistent findings in the cognitive literature of ADHD (4, 5, 12, 13, 15–17). Increased RTV in particular is the most replicated finding in individuals with ADHD compared to TD (12, 14, 16, 17); it has frequently been related to excessively slow RTs in individuals with ADHD (14) and is associated with ADHD diagnosis and symptoms (14, 15), with some considering it to underlie behavioral inattention in ADHD (16, 60). RTV has furthermore been shown to be specific to ADHD rather than ASD traits (13, 61).

The changes for CV in this study between guanfacine and placebo were very large with a Cohen’s d of 1.3 which would be clinically relevant. The difference in MRT between the TD and the participants with ADHD on placebo were ~0.6 SD which is larger than established thresholds for changes in measures of clinical significance of more than 0.5 SD (62).

Meta-analytic evidence shows that poor processing speed in children with ADHD is strongly negatively correlated with academic skills and with clinical problems such as poorer adaptive skills, increased self-reported anxiety and overestimating social competence (63). RTV likewise has been associated with poorer academic performance (64), motor development (65), and motor timing (21, 66) in children with ADHD. In line with a role in attention and self-control, on a neural level, RTV has been associated with smaller white matter tracts in the late developing fronto-parietal attention (superior longitudinal fasciculus) and cortico-thalamic cognitive control tracts and reduced activation in fronto-striatal and superior temporal activation in children and adults with ADHD (18, 67–70), suggesting an association with immature brain development in attention and self-control regions and networks. Furthermore, increased RTV has been shown to be a behavioral index of increased mind-wandering in adults with ADHD (71–73), and has been associated with abnormalities in “switching off” the neural correlates of mind-wandering, the default mode network (DMN) (74) during task performance in children and adults with ADHD (19, 20).

The findings that acute lisdexamfetamine administration improved the two key cognitive dysfunction measures of RT and CV relative to placebo extends findings of chronic lisdexamfetamine improving RT, RTV and attentional measures in children and adolescents with ADHD (34–36). They extend findings of methylphenidate consistently improving RTV and RT in children with ADHD (22, 29–31), with meta-analytic fMRI evidence showing that this may be underpinned neurofunctionally by MPH-induced deactivation of frontal areas of the DMN which is associated with RTV in people with ADHD (75).

The detrimental effects of acute guanfacine on CV relative to placebo and lisdexamfetamine are concerning given the above discussed key role of RTV in ADHD cognition. The weaker non-significant medication effects on CPT omission errors were also partly driven by guanfacine (i.e., guanfacine deteriorating them and lisdexamfetamine improving them). Together, they could suggest that guanfacine may have a negative effect on attention measures in ADHD. The findings extend evidence for detrimental effects of longer-term guanfacine use on WM in a much larger sample of 172 children with ADHD (43). They also extend evidence of no acute or chronic effects of guanfacine on cognitive performance in children and adults with ADHD (39–42). Together, this suggests that guanfacine, while improving ADHD symptoms relative to placebo (39–41, 43, 44), may not be an optimal drug to improve specific attention measures.

It is unclear why and how guanfacine increased CV. It is possible that the sedating effect of guanfacine (27), due to documented effects of sedation on attention (76, 77) may have caused poorer performance in response consistency. Out of six studies testing effects of mostly longer-term guanfacine effects on cognition, four reported sedative symptoms (39, 40, 43) or fatigue (44) while two other studies did not record or report them (39, 42). While only one of these studies reported reduced cognitive performance in a WM task (43), impaired attention (as well as memory, working memory, reaction time and psychomotor function) has been observed after clonidine in neurotypical adults (78, 79), which similarly to guanfacine, is also an α2-adrenergic receptor agonist and can cause sedation. However, in this study we did not find that guanfacine had more side effects than lisdexamfetamine, not even on the sedation-related items of drowsiness or fatigue. On the contrary, parents/carers reported more adverse effects after lisdexamfetamine compared to placebo and guanfacine. It is noteworthy, however, that we did not ask the children with ADHD but their parents only and we recorded drowsiness and fatigue, instead of sedation.

Decreased arousal could be another possible underlying mechanism of impaired CV under guanfacine, since hypo-arousal has been associated with RTV (80, 81). There is evidence that guanfacine decreases arousal in animal models (82) and people with addiction (83), and decreases physiological parameters that are related to arousal like blood pressure and pulse in children with ADHD (40, 84). A reduction in arousal could potentially exacerbate attentional lapses and increase RTV/CV.

Our findings of improved MRT and CV with lisdexamfetamine, but worsened CV with guanfacine may also be interpreted in light of differential effects on brain activation observed in fMRI studies comparing stimulants and non-stimulants. Thus, in our four fMRI studies comparing the effects of single dosages of methylphenidate and atomoxetine versus placebo on brain activation in children and adolescents with ADHD (45, 85–87), we found that reduced activation in frontal, parietal, temporal, and insular-striatal regions during cognitive tasks can be increased (and even normalized) by both stimulant and non-stimulant medications. Specifically, during inhibitory control tasks, both methylphenidate and atomoxetine enhanced activation in the right inferior parietal/superior temporal areas and left ventrolateral prefrontal cortex (VLPFC), but only methylphenidate reduced activation in the right VLPFC and cerebellum (85). During working memory, atomoxetine uniquely increased activation in the right dorsolateral prefrontal cortex (DLPFC) compared to methylphenidate, whereas methylphenidate upregulated activation in the left inferior frontal cortex (IFC), suggesting drug-specific laterality effects (while both drugs enhanced fronto-temporo-striatal activation) (45). Furthermore, both methylphenidate and atomoxetine normalized reduced activation relative to TD in parietal, temporal, precuneus/posterior cingulate, and striato-insular regions during sustained attention with drug specific upregulation of ventral fronto-temporal regions including the left VLPFC for methylphenidate (86) and both drugs normalized activations in the right VLPFC, insula, and striatal regions during timing tasks (87). It thus appears that stimulants compared to non-stimulants have more pronounced activation and normalization effects on areas of the ventral attention network (in particular inferior frontal regions) which could potentially be the underlying mechanisms by which lisdexamfetamine has stronger effects on RTV, sustained attention and time estimation which are mediated by these inferior prefrontal regions (19, 88–92).

Furthermore, a recent fMRI comparative meta-analysis of 29 fMRI studies of stimulants (20 studies) and of non-stimulants (9 studies) showed overlapping but also differential drug effects. While both drugs normalized left SMA activation which overlapped with regions of the central executive control, only non-stimulants normalized regions of the ventral attention and limbic systems (i.e. left superior frontal and mid-cingulate cortices and left amygdala). These differential positive effects on the ventral attention system may be responsible for the improvement in RTV and MRT with lisdexamfetamine (as well as improvements in sustained attention and time discrimination) (93). Non-stimulants on their own reduced left prefrontal and caudate activations which could potentially be an underlying mechanism for the here observed worsening of cognitive attention measures of RTV (and eventual sustained attention) with guanfacine. However, out of the nine meta-analyzed fMRI studies that examined non-stimulants, eight studies tested atomoxetine and only one study tested guanfacine. Future research should therefore further examine the differential brain function effects of stimulant and non-stimulant drugs, especially the neurofunctional mechanisms of action of guanfacine that are so far poorly understood.

The worsening effects of guanfacine on CV, while concerning, need, however, be considered in light of the relatively small subject numbers. Nevertheless, our study adds to the majority of previous studies showing either no (39–42) or negative effects (43) of guanfacine on cognition in children with ADHD, with only a single very small numbered study in 17 adults with ADHD showing cognitive improvement in interference inhibition (44). Future studies should use larger samples to test effects of guanfacine on RTV, considering its real-life implications.

We also showed medication effects on some attentional and timing measures with moderate to large effect sizes which were, however, slightly above the threshold of significance. Thus, we found that lisdexamfetamine numerically improved time discrimination and CPT omission errors, the latter of which were numerically worsened with guanfacine. These findings could extend evidence for acute and chronic methylphenidate administration significantly improving timing (66, 94) and attention functions in children with ADHD including the same tasks (22, 29–31).

None of the drugs improved inhibitory measures. While this is in line with the majority of the previous negative findings of guanfacine effects on these measures (39, 41, 42), for lisdexamfetamine, some studies of longer-term use found improvement in inhibitory measures (34, 36) while others did not (38). This was also unexpected as methylphenidate both in acute and longer-term dosage has been shown to improve inhibitory measures (17, 22, 29–31). It is possible that the acute dosage is less efficient in improving cognitive measures than the longer-term dosage, or that the study was underpowered to show lisdexamfetamine effects on inhibitory measures.

## 5 Limitations

This study has several limitations. First, the sample size of 22 children and adolescents with ADHD was powered for fMRI and was therefore relatively small for a neurocognitive study and might explain the non-significance of some findings that were of medium (time discrimination) to large effects sizes (CPT omission errors). Second, the study design may have favored lisdexamfetamine as there was a discrepancy between the t-max of lisdexamfetamine (3.5 – 4.5h) and guanfacine (5h), with the t-max of guanfacine not reached during the neurocognitive testing, which took place 3h after medication administration (the time schedule was tailored toward the fMRI 4.5h after medication administration). Furthermore, guanfacine takes longer to show behavioral effects than lisdexamfetamine (several weeks vs 24h) and hence comparative studies on acute effects of stimulants versus non-stimulants may favor stimulant medication. Third, the age range of participants (8–20 years) was relatively broad. While age-related differences cannot be completely ruled out, the within-subject design of the study, with participants serving as their own controls likely mitigates potential confounding effects of age. Fourth, we did not exclude comorbidities like ASD which may have confounded the findings. However, our regression analysis did not demonstrate a contribution of ASD diagnosis or traits on the main findings. Fifth, although our analysis did not find significant order effects, we cannot completely rule them out, given that our analysis included six different orders within a relatively small sample of 22 participants and the statistical power to detect such effects was limited.

## 6 Conclusion

This is the first study comparing differential acute effects of GXR and lisdexamfetamine, relative to placebo. Lisdexamfetamine improved RT and CV, extending consistent findings of positive effects on these measures with methylphenidate. Guanfacine, however, showed no improvement in any measure, with concerning deterioration relative to lisdexamfetamine and placebo on CV. The findings of lisdexamfetamine improving and guanfacine deteriorating one of the most consistently replicated performance measures of ADHD, i.e., response consistency, in youth with ADHD, are clinically relevant and encourage future research, which should implement larger samples.

## Data Availability

The raw data supporting the conclusions of this article will be made available by the authors, without undue reservation.
