# Supplementary material for: Differential effects of a single dose of Lisdexamfetamine and Guanfacine on cognitive function in children with ADHD
Source: Front Psychiatry. 2025 Nov 3;16:1676472. doi: 10.3389/fpsyt.2025.1676472 (PMC12622228; doi:10.3389/fpsyt.2025.1676472)
Supplement: Supplementary file 1 [file Supplementaryfile1.docx]

# **Supplemental data A: Schematic displays of neurocognitive tasks**

**1) Go/No-Go Task**

This is a selective motor response inhibition task measuring response inhibition and selective attention (Penadés et al., 2007; Rubia et al., 2001). In this task, participants are asked to execute a motor response as fast as possible using the left/right arrow buttons on the keyboard to high frequent Go signals (i.e., spaceships of 300ms duration; 73.3%) and to inhibit their motor response to infrequent No-Go signals (i.e., enemy planets of 300ms duration; 26.7%). There are 300 trials in total, 220 Go trials and 80 No-Go trials. All signals appear in the middle of the screen followed by a blank screen of 1300ms. The task is divided into two subtests of 2min and 32s each, blocked for a right- and a left-handed response respectively, to increase the prepotent response tendency. The task is identical in number of trials, except that all spaceships point to the right in the right-handed response subtest and to the left in the left-handed response subtest. The total task duration is 5 minutes and 4 seconds. The dependent variable for this task is the probability of inhibition (%) to the No-Go stimuli.


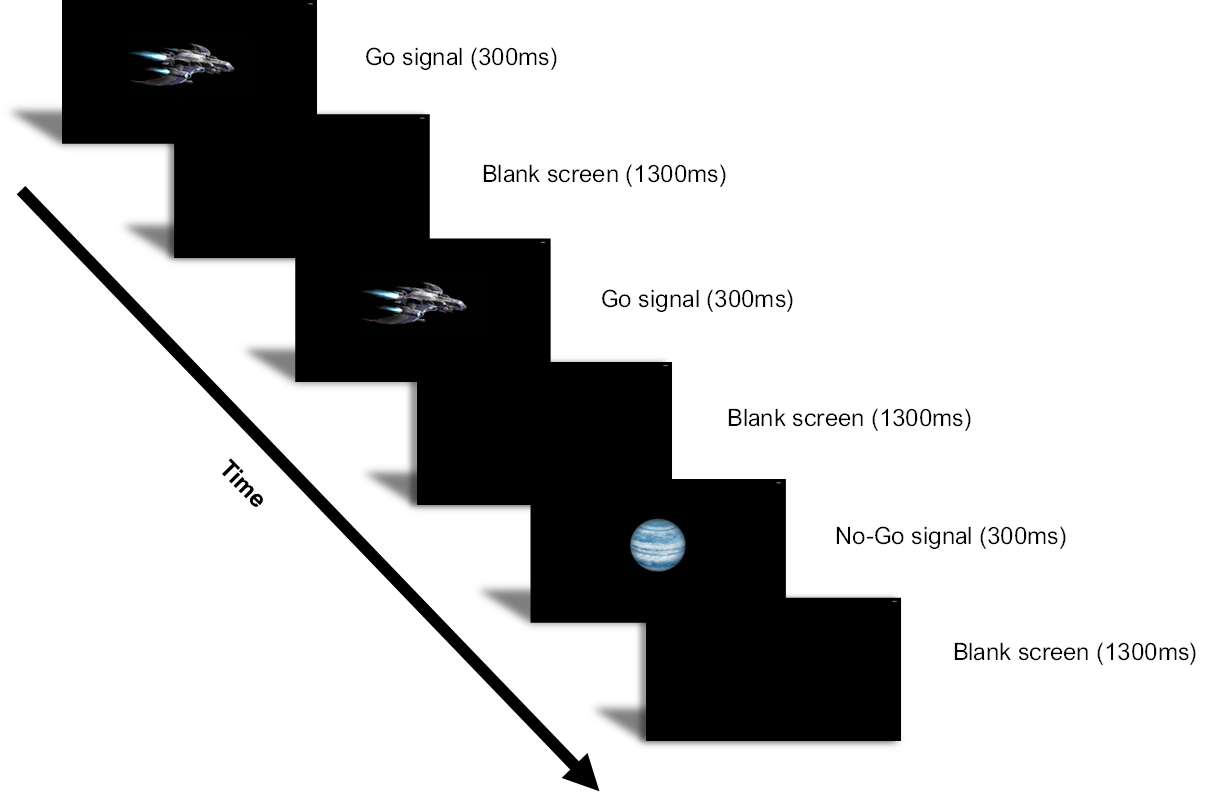


**Figure 1. Schematic display of the right-handed response trials of the Go/No-Go task. Subjects are instructed to respond to every Go signal and inhibit their motor responses to the No-Go signals.**

**2) Simon Task**

The Simon interference inhibition task measures interference inhibition and selective attention (Penadés et al., 2007; Rubia et al., 2007). In this task, yellow arrows pointing left or right appear on the left or right side of the screen; participants respond by pressing either the left or the right arrow key, according to iconic information provided by the stimulus (where the arrows are pointing). On 72.7% of the trials the arrows are congruent (i.e., the direction of they point, and the side of the screen are congruent). In 27.3% of the trials the arrows that are pointing right appeared on the left side of the screen or the arrows pointing left appeared on the right side of the screen, which are spatially incompatible cues that interfere with the primary intended action. Participants have to override the prepotent response tendency to incorrectly respond to these spatially incompatible cues. There are 220 trials in total (160 congruent, 60 incongruent). All stimuli appeared with a duration of 300ms followed by a blank screen of 1700ms, resulting in an inter-trial-interval (ITI) of 2s. The total task duration is 5 minutes and 8 seconds. The dependent variable for this task is the Simon reaction time effect (mean reaction time to congruent trials subtracted from mean reaction time to incongruent trials).


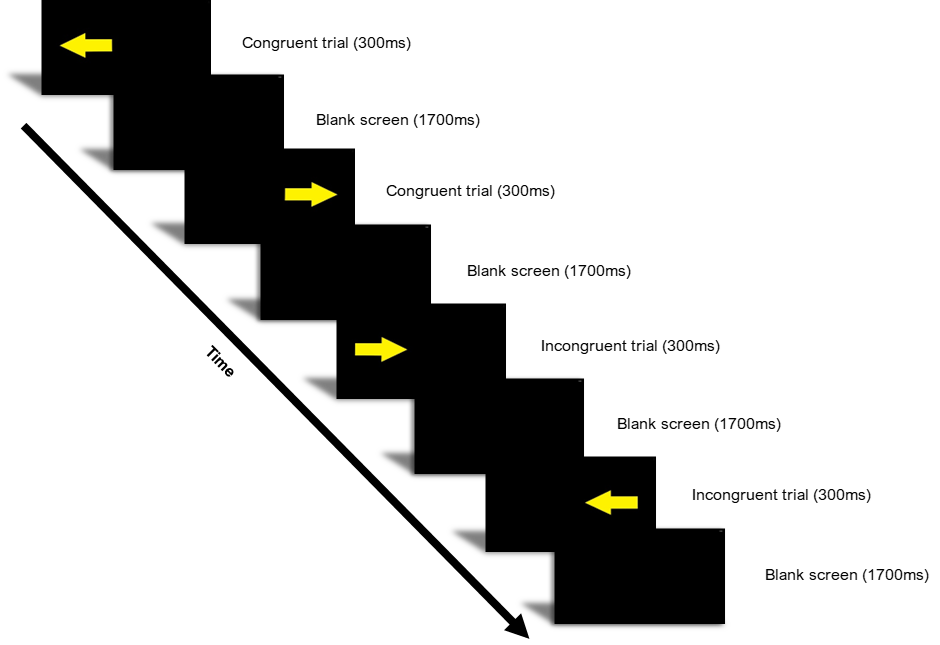


**Figure 2. Schematic display of the Simon Interference Inhibition task. Subjects are instructed to respond using the left arrow key for left-pointing arrows and the right arrow key for right-pointing arrows.**

**3) Continuous Performance Task**

The Continuous Performance task (CPT) measures target detection, sustained and selective attention (Penadés et al., 2007; Rubia et al., 2007). Participants are presented with a string of letters (alphabet A to L) and have to ignore all letters except for specific target letter combinations ("A" followed by "X" or "A" followed by "O"). Participants have to respond using the left arrow key for every “A-X” and the right arrow key for every “A-O" sequence. The number of correct responses is displayed and fed back to participants visually throughout the task by two rising score-bars (red for A-X and blue for A-O) on the right-hand side of the screen. For every three correct hits, a reward bar with 10 units is filled. There are 480 trials in total, with 60 target trials (30 A-X and 30 A-O targets). The duration of the letters is 300ms followed by a blank screen, with a mean ITI of 900ms. The total task duration is 8 minutes. Dependent variables for this task are the percentage of omission errors (i.e., missed detection to target trials) and commission errors (i.e., incorrect detection to nontarget items, false hits).


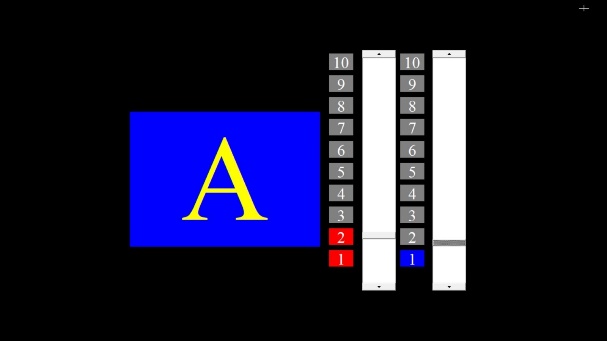


**Figure 3. Schematic display of the Continuous Performance task. Subjects are instructed to respond using the left arrow key for every “A-X” and the right arrow key for “A-O”; and ignore other letters.**

**4) Time Discrimination Task**

The Time Discrimination task measures the participant’s ability to discriminate between time intervals that differ by several hundreds of milliseconds (Rubia et al., 2007). Participants are presented with 60 pairs of circles (red and green) appearing consecutively with no interspersed pause and in random order, with the red circle consistently presented on the right side and the green circle on the left side of the computer screen. One of these circles is randomly presented for a standard duration of 1000ms, and the comparison circle for either 1300ms, 1400ms, or 1500ms. After presenting a standard interval and a comparison interval, there is a blank screen for 2.1s in which participants decide which circle stayed on the screen for the longest time and responded by pressing the right arrow button if they think the right, red circle lasted longest, or pressing the left arrow key if they believe the green, left circle lasted the longest. Children are instructed to discriminate the time by counting to ensure a consistent and homogenous performance strategy. The total task duration is 4.5 minutes. The dependent variable for this task is the percentage of time discrimination errors.


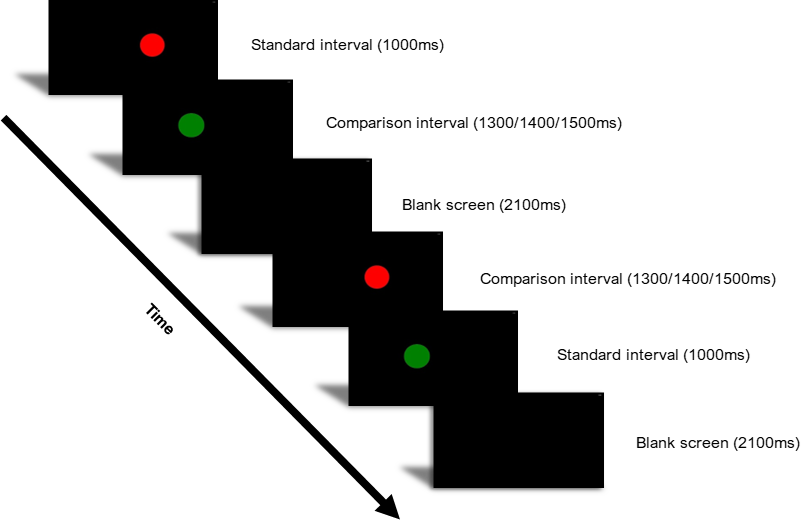


**Figure 4. Schematic display of the Time Discrimination task. Red and green circles appear consecutively in random order; subjects have to decide which of the two circles stayed on the screen for the longest time.**

**5) Mackworth Clock Task**

This task is an adaptation (Lichstein et al., 2000) based on the original Mackworth Clock Task (Mackworth, 1948) measuring sustained attention and vigilance on the detection of signals that are difficult to identify (see <https://www.psytoolkit.org/>; Stoet, 2010, 2017). Participants are asked to pay attention to a clock hand that rotated clockwise in the middle of the screen. When the clock hand does a double jump skipping a second (10% of trials, i.e. 40 of 400 trials), participants have to press the space bar immediately within 1s. Participants receive an error signal (red light at the centre of the clock hand) when the motor response is made in the absence of the double jumps of the clock hand (commission errors) or when they fail to detect the double jumps of the clock hand (omission errors). Participants receive positive feedback (green light) when the motor response is executed correctly. The total task duration is 7 minutes. Dependent variables for this task are: the percentage of omission errors (i.e., missed detection to target trials) and percentage of commission errors (i.e., incorrect detection to nontarget items, false hits).


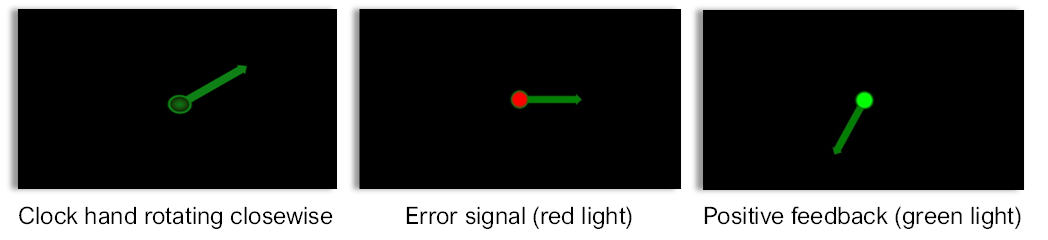


**Figure 5. Schematic display of the Mackworth Clock task. Subjects have to respond to every double jump that skips a second on the clock hand.**

# **Supplemental data B: Formulas for calculation of composite scores**

Additional measures were component measures of Mean Reaction Time (MRT) in ms, Coefficient of variation (CV), and percentage of premature responses in percentage across the Go/No-Go, Simon and CPT tasks (see Lam et al., 2022). The MRT composite measure across tasks is adjusted by the number of response trials (i.e., go/congruent/target) in each of the 3 tasks. The Coefficient of variation (CV) is a measure of relative RTV and reflects intraindividual variability in reaction times (RT) adjusted for their mean, i.e. it is defined as the ratio of the standard deviation to the mean: CV = SD/Mean (Everitt and Skrondal, 2010, p. 58). This composite measure of CV across the 3 tasks is adjusted for the number of go/congruent/target trials in each of the 3 tasks. Premature responses are defined as responses made before the stimulus appears (200ms before stimulus appearance) or before it has been registered (100ms after stimulus - considered too short to be an average normal reaction time), reflecting behavioural impulsiveness in ADHD (Rubia et al., 2007). Like the other measures, premature responses are also adjusted by the number of trials for each task. Equations to calculate the composite scores are shown in equations 1, 2, 3 (see also Lam et al., 2022).

**Equation 1. Equation for Mean Reaction Time Composite.**

$$\frac{\left( N_{Go}\times MRT_{Go} \right)+\left( N_{Cong}\times MRT_{Cong} \right)+\left( N_{CPTt}\times MRT_{CPTt} \right)}{N_{Go}+N_{Cong}+N_{CPTt}}$$

N_Go_, number of Go trials; MRT_Go_, mean RT of Go trials; N_Cong_, number of Simon congruent trials; MRT_Cong_, mean RT of Simon congruent trials; N_CPTt_, number of CPT target trials; MRT_CPTt_, mean RT of CPT target trials.

**Equation 2. Equation for Coefficient of Variation Composite.**

$$\frac{\left( N_{Go}\times SDRT_{Go} \right)+\left( N_{Cong}\times SDRT_{Cong} \right)+\left( N_{CPTt}\times SDRT_{CPTt} \right)}{\left( N_{Go}\times MRT_{Go} \right)+\left( N_{Cong}\times MRT_{Cong} \right)+\left( N_{CPTt}\times MRT_{CPTt} \right)}$$

SD, standard deviation; N_Go_, number of Go trials; SDRT_Go_, SD of RT of Go trials; N_Cong_, number of Simon congruent trials; SDRT_Cong_, SD of RT of Simon congruent trials; N_CPTt_, number of CPT target trials; SDRT_CPTt_, SD of RT of CPT target trials.

**Equation 3. Equation for Premature Response Composite.**

$$\frac{\left( N_{GNG}\times PREM_{GNG} \right)+\left( N_{Simon}\times PREM_{Simon} \right)+\left( N_{CPT}\times PREM_{CPT} \right)}{N_{GNG}+N_{Simon}+N_{CPT}}$$

N_Go/No-Go_, number of all _Go/No-Go_ trials; PREM_Go/No-Go_, number of premature responses in all Go/No-Go task trials; N_Simon_, number of all Simon task trials; PREM_Simon_, number of premature responses in Simon task trials; N_CPT_, number of all CPT task trials; PREM_CPT_, number of premature responses in CPT task trials.

# **Supplemental data C: Summary table of means (SD), Friedman tests and post-hoc Wilcoxon signed rank tests of medication effects on cognitive performance and adverse effects**

| **Cognitive variables** | **N** | **GFC**  **Mean (SD)** | **LDX**  **Mean (SD)** | **Placebo**  **Mean (SD)** | **Friedman Chi-Square** | **df** | **P value** | **Kendall’s W** | **Post-hoc Wilcoxon Signed-rank test** |
| --- | --- | --- | --- | --- | --- | --- | --- | --- | --- |
| Go/No-Go PI (%) | 22 | 60.1 (22.6) | 65.1 (22.0) | 66.1 (19.00) | 2.138 | 2 | 0.343 | 0.049 | - - - |
| Simon RT effect (ms) | 22 | 53.4 (26.2) | 56.8 (29.7) | 52.9 (33.8) | 1.091 | 2 | 0.580 | 0.025 | - - - |
| CPT OM errors (%) | 22 | 12.0 (10.8) | 6.8 (7.8) | 8.70 (8.70) | 4.078 | 2 | 0.130 | 0.097 | - - - |
| CPT COM errors (%) | 22 | 1.5 (2.7) | 1.0 (1.1) | 0.9 (1.0) | 3.410 | 2 | 0.182 | 0.081 | - - - |
| MCT OM errors (%) | 20 | 28.0 (20.3) | 22.1 (18.0) | 27.3 (20.6) | 3.795 | 2 | 0.150 | 0.095 | - - - |
| MCT COM errors (%) | 20 | 2.5 (2.1) | 1.9 (1.7) | 2.0 (1.9) | 1.014 | 2 | 0.602 | 0.025 | - - - |
| Time discrimination errors(%) | 21 | 26.8 (19.5) | 21.0 (17.2) | 28.1 (23.6) | 4.837 | 2 | 0.089 | 0.110 | - - - |
| MRT composite (ms) | 22 | 389.2 (60.8) | 370.3 (54.1) | 392.7 (57.8) | 16.667 | 2 | 0.001 | 0.397 | GFC vs LDX: (p=0.005)*^1^  GFC vs PLA: (p=0.466)*^1^  LDX vs PLA: (p=0.005)*^1^ |
| CV composite | 22 | 0.3 (0.1) | 0.2 (0.1) | 0.2 (0.1) | 13.714 | 2 | <0.001 | 0.327 | GFC vs LDX (p=0.003)*^1^  GFC vs PLA: (p=0.017)*^1^  LDX vs PLA: (p=0.005)*^1^ |
| PREM composite | 22 | 0.8 (1.4) | 0.6 (1.5) | 0.5 (1.1) | 3.973 | 2 | 0.137 | 0.095 | - - - |
| Adverse effects questionnaire - Total score | 21 | 2.71 (3.09) | 8.52 (7.02) | 1.76 (1.97) | 20.079 | 2 | <0.001 | 0.478 | GFC vs LDX: (p=0.004)^*1^  GFC vs PLA: (p= 0.159)^*1^  LDX vs PLA: (p=0.003)^*1^ |

significant at p = 0.05; ^1^ = FDR corrected; GFC = Guanfacine; LDX = Lisdexamfetamine; PI= Probability of inhibition; RT= Reaction time; CPT= Continuous Performance Task; MCT= Mackworth Clock Task; MRT= Mean reaction time; CV= Coefficient of variation; PREM= Premature responses

Significant Friedman tests confirmed intra-subject medication effects in the ADHD group for MRT (p= 0.001) and CV (p <0.001) and at a weaker level, short of significance findings for time discrimination (p= 0.089). Moreover, FDR-corrected post-hoc Wilcoxon Signed-Ranks tests confirmed that lisdexamfetamine improved MRT and CV relative to placebo (MRT: Z= -2.972, p= 0.005; CV: Z= -2.728, p= 0.005) and compared to guanfacine (MRT: Z= -2.728, p= 0.005; CV: Z=-3.702, p=0.00), while guanfacine worsened CV relative to placebo (Z= 2.138, p= 0.017) and lisdexamfetamine (Z= 2.728, p=0.003). Friedman tests showed also significant effects for total adverse effects (p= 0.001). Post-hoc Wilcoxon Signed-Ranks tests showed that parents/carers of youth with ADHD reported more total adverse effects in their children following lisdexamfetamine relative to placebo (Z= 3.701, p= 0.003) and relative to guanfacine (Z= 2.958, p= 0.004), while guanfacine and placebo did not differ (Z= 1.409, p= 0.159) (all p-values were FDR-corrected).

# **Supplemental data D: Summary table of means (SD), repeated measures ANOVA and post hoc t-tests results of medication effects on cognitive performance and adverse effects without the two participants that chewed the guanfacine tablets**

| **Dependent Variable** | **N** | **GFC**  **Mean (SD)** | **LDX**  **Mean (SD)** | **Placebo**  **Mean (SD)** | **F** | **df** | **P value** | **Cohen’s d** | **Post hoc t-tests** |
| --- | --- | --- | --- | --- | --- | --- | --- | --- | --- |
| Go/No-Go PI all (%) | 20 | 60.06 (22.14) | 63.69 (22.38) | 64.31 (18.85) | 1.14 | 2 | 0.329 | 0.49 | - - - |
| Simon RT effect (ms) | 20 | 52.53 (27.31) | 57.47 (30.52) | 53.57 (33.57) | 0.20 | 1.6*^1^ | 0.764*^1^ | 0.20 | - - - |
| CPT OM errors (%) | 19 | 11.58 (10.52) | 7.54 (7.90) | 9.39 (8.88) | 2.07 | 2 | 0.141 | 0.68 | - - - |
| CPT COM errors (%) | 19 | 1.64 (2.81) | 1.07 (1.13) | 0.96 (0.97) | 1.18 | 1.3*^1^ | 0.302*^1^ | 0.51 | - - - |
| MCT OM errors (%) | 18 | 24.58 (18.36) | 21.81 (19.00) | 26.67 (21.37) | 0.69 | 2 | 0.508 | 0.40 | - - - |
| MCT COM errors (%) | 18 | 2.16 (1.85) | 1.93 (1.82) | 2.05 (1.98) | 0.24 | 2 | 0.834 | 0.21 | - - - |
| Time discrimination errors (%) | 20 | 25.42 (19.76) | 20.33 (17.22) | 28.42 (24.53) | 2.79 | 1.5*^1^ | 0.092*^1^ | 0.77 | - - - |
| MRT composite (ms) | 19 | 383.97 (61.47) | 366.56 (55.49) | 388.94 (59.71) | 6.90 | 2 | 0.003 | 1.24 | GFC vs LDX: d= 0.64 (p=0.009)*^2^  LDX vs PLA: d= -0.77 (p=0.006)*^2^ |
| CV composite | 19 | 0.25 (0.07) | 0.22 (0.08) | 0.23 (0.07) | 6.34 | 1.4*^1^ | 0.011*^1^ | 1.19 | GFC vs LDX: d= 1.33 (p=0.003)*^2^  GFC vs PLA: d= 0.39 (p= 0.078)*^2^  LDX vs PLA: d= -0.39 (p=0.085)*^2^ |
| PREM composite | 19 | 0.78 (1.49) | 0.66 (1.58) | 0.57 (1.13) | 0.59 | 1.1*^1^ | 0.473*^1^ | 0.36 | - - - |
| Adverse effects questionnaire - Total score | 19 | 2.00 (2.05) | 8.58 (7.39) | 1.58 (1.92) | 15.2 | 1.3*^1^ | <0.001*^1^ | 1.83 | GFC vs LDX: d= -0.86 (p=0.002)*^2^  GFC vs PLA: d= 0.14 (p= 0.275)*^2^  LDX vs PLA: d= 1.00 (p= 0.002)*^2^ |

significant at p = 0.05; ^1^ = Greenhouse-Geisser correction; ^2^ = FDR corrected; GFC = Guanfacine; LDX = Lisdexamfetamine; PI= Probability of inhibition; RT= Reaction time; CPT= Continuous Performance Task; MCT= Mackworth Clock Task; MRT= Mean reaction time; CV= Coefficient of variation; PREM= Premature responses

After excluding the 2 participants who chewed the guanfacine tablets, intra-subject main effects using ANOVA remained significant for MRT (p= 0.003) and CV (p= 0.011) and at weaker level, short of significance time discrimination (p=0.092). FDR-corrected post-hoc test results also remained similar: lisdexamfetamine still improved MRT relative to placebo (t(df=18)= -3.356, p= 0.006;) and guanfacine (t(df=18)= -2.792, p= 0.009) and CV compared to guanfacine (t(df=18)= -5.827, p=0.003) and at weaker level below the threshold of significance compared to placebo (t(df=18)= -1.428, p= 0.085). The effect of guanfacine increasing CV compared to placebo changed to below the threshold of significance (t(df=18)= 1.707, p= 0.078). ANOVA also showed significant effects for total adverse effects (p< 0.001). Post-hoc t-tests showed that parents/carers of youth with ADHD reported more total adverse effects in their children following lisdexamfetamine relative to placebo (t(df=18)= 4.379, p= 0.002) and relative to guanfacine (t(df=18)= -3.768, p= 0.002), while guanfacine and placebo did not differ (t(df=18)= 0,611, p= 0.275) (all p-values were FDR-corrected).

# **Supplemental data E: Summary table of means (SD), Friedman tests and post-hoc Wilcoxon signed rank tests of medication effects on cognitive performance and adverse effects without the two participants that chewed the guanfacine tablets**

| **Cognitive variables** | **N** | **GFC**  **Mean (SD)** | **LDX**  **Mean (SD)** | **Placebo**  **Mean (SD)** | **Friedman Chi-Square** | **df** | **P value** | **Kendall’s W** | **Post-hoc Wilcoxon Signed-rank test** |
| --- | --- | --- | --- | --- | --- | --- | --- | --- | --- |
| Go/No-Go PI all (%) | 20 | 60.06 (22.14) | 63.69 (22.38) | 64.31 (18.85) | 0.937 | 2 | 0.626 | 0.020 | - - - |
| Simon RT effect (ms) | 20 | 52.53 (27.31) | 57.47 (30.52) | 53.57 (33.57) | 1.200 | 2 | 0.549 | 0.030 | - - - |
| CPT OM errors (%) | 19 | 11.58 (10.52) | 7.54 (7.90) | 9.39 (8.88) | 2.314 | 2 | 0.314 | 0.061 | - - - |
| CPT COM errors (%) | 19 | 1.64 (2.81) | 1.07 (1.13) | 0.96 (0.97) | 1.861 | 2 | 0.394 | 0.049 | - - - |
| MCT OM errors (%) | 20 | 24.58 (18.36) | 21.81 (19.00) | 26.67 (21.37) | 2.400 | 2 | 0.301 | 0.067 | - - - |
| MCT COM errors (%) | 20 | 2.16 (1.85) | 1.93 (1.82) | 2.05 (1.98) | 0.769 | 2 | 0.681 | 0.021 | - - - |
| Time discrimination errors (%) | 20 | 25.42 (19.76) | 20.33 (17.22) | 28.42 (24.53) | 5.026 | 2 | 0.081 | 0.126 | - - - |
| MRT composite (ms) | 19 | 383.97 (61.47) | 366.56 (55.49) | 388.94 (59.71) | 13.579 | 2 | 0.001 | 0.357 | GFC vs LDX: (p=0.012)*1  GFC vs PLA: (p=0.344)*1  LDX vs PLA: (p=0.009)*1 |
| CV composite | 19 | 0.25 (0.07) | 0.22 (0.08) | 0.23 (0.07) | 10.526 | 2 | 0.005 | 0.277 | GFC vs LDX: (p=0.003)*1  GFC vs PLA: (p=0.042)*1  LDX vs PLA: (p=0.011)*1 |
| PREM composite | 19 | 0.78 (1.49) | 0.66 (1.58) | 0.57 (1.13) | 2.358 | 2 | 0.308 | 0.062 | - - - |
| Adverse effects questionnaire - Total score | 19 | 2.00 (2.05) | 8.58 (7.39) | 1.58 (1.92) | 18.889 |  | <0.001 | 0.497 | GFC vs LDX: p= 0.002^*1^  GFC vs PLA: p= 0.196^*1^  LDX vs PLA: p= 0.002^*1^ |

significant at p = 0.05; ^1^= FDR corrected; GFC = Guanfacine; LDX = Lisdexamfetamine; PI= Probability of inhibition; RT= Reaction time; CPT= Continuous Performance Task; MCT= Mackworth Clock Task; MRT= Mean reaction time; CV= Coefficient of variation; PREM= Premature responses

After excluding the 2 participants who chewed the guanfacine tablets significant Friedman tests confirmed intra-subject medication effects in the ADHD group for MRT (p= 0.001) and CV (p= < 0.005) and at a weaker level, short of significance for time discrimination (p= 0.081). Moreover, FDR-corrected post-hoc Wilcoxon Signed-Ranks tests confirmed that lisdexamfetamine improved MRT and CV relative to placebo (MRT: Z= -2.736, p= 0.009; CV: Z= -2.455, p= 0.011) and compared to guanfacine (MRT: Z= -2.415, p= 0.012; CV: Z= -3.416, p= 0.003), while guanfacine worsened CV relative to placebo (Z= 1.730, p=0.042) and lisdexamfetamine (Z= 3.461, p= 0.003). Friedman tests showed also significant effects for total adverse effects (p <0.001). Post-hoc Wilcoxon Signed-Ranks tests showed that parents/carers of youth with ADHD reported more total adverse effects in their children following lisdexamfetamine relative to placebo (Z= 3.469, p= 0.002) and relative to guanfacine (Z= 3.048, p= 0.002), while guanfacine and placebo did not differ (Z= 0.856, p= 0.196) (all p-values were FDR-corrected).

# **References**

Everitt, B.S., Skrondal, A., 2010. The Cambridge Dictionary of Statistics (4^th^ ed.). United States of America by Cambridge University Press, New York 480.

Lam, S.-L., Criaud, M., Lukito, S., Westwood, S.J., Agbedjro, D., Kowalczyk, O.S., Curran, S., Barret, N., Abbott, C., Liang, H., Simonoff, E., Barker, G.J., Giampietro, V., Rubia, K., 2022. Double-Blind, Sham-Controlled Randomized Trial Testing the Efficacy of fMRI Neurofeedback on Clinical and Cognitive Measures in Children With ADHD. AJP 179, 947–958. https://doi.org/10.1176/appi.ajp.21100999

Lichstein, K.L., Riedel, B.W., Richman, S.L., 2000. The Mackworth Clock Test: A Computerized Version. The Journal of Psychology 134, 153–161. https://doi.org/10.1080/00223980009600858

Mackworth, N.H., 1948. The Breakdown of Vigilance during Prolonged Visual Search. Quarterly Journal of Experimental Psychology 1, 6–21. https://doi.org/10.1080/17470214808416738

Penadés, R., Catalán, R., Rubia, K., Andrés, S., Salamero, M., Gastó, C., 2007. Impaired response inhibition in obsessive compulsive disorder. European Psychiatry 22, 404–410. https://doi.org/10.1016/j.eurpsy.2006.05.001

Rubia, K., Smith, A., Taylor, E., 2007. Performance of Children with Attention Deficit Hyperactivity Disorder (ADHD) on a Test Battery of Impulsiveness. Child Neuropsychology 13, 276–304. https://doi.org/10.1080/09297040600770761

Rubia, K., Taylor, E., Smith, A.B., Oksannen, H., Overmeyer, S., Newman, S., 2001. Neuropsychological analyses of impulsiveness in childhood hyperactivity. Br J Psychiatry 179, 138–143. https://doi.org/10.1192/bjp.179.2.138
